# Supplementary material for: Cadmium and Lead Tolerance of Filamentous Fungi Isolated from Contaminated Mining Soils
Source: Biology (Basel). 2025 Jun 12;14(6):688. doi: 10.3390/biology14060688 (PMC12189132; doi:10.3390/biology14060688)
Supplement: Supplementary file 1 [file biology-14-00688-s001.zip › biology-3661344-supplementary.pdf]

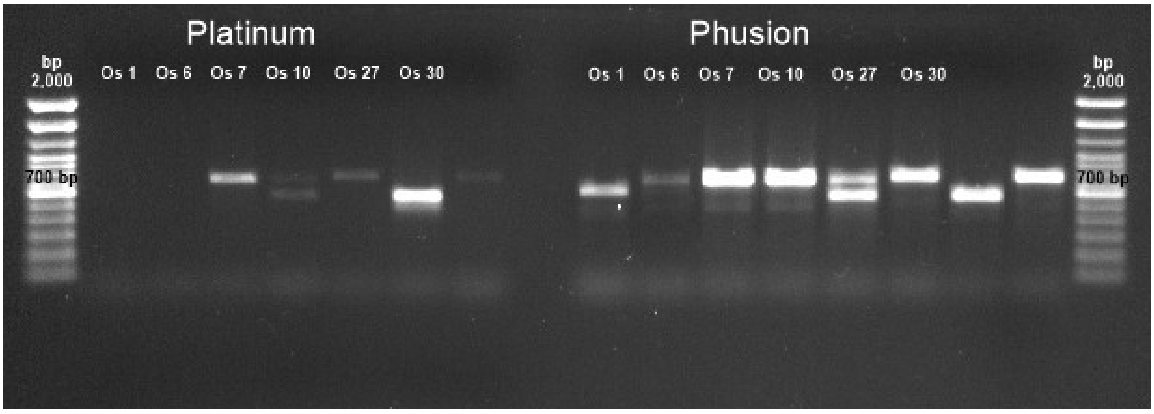


Fig. S1. Optimization of ITS amplification using dual Taq polymerases targeting ITS4 and ITS5 primers for improved molecular identification of fungal isolates. Platinum™ Taq DNA Polymerase. Phusion™ High-Fidelity DNA Polymerase.

Table S1. Comparison of maximum tolerated concentrations of heavy metals by filamentous fungi in this study and previous reports.

| Fungal Species | Metal | Max. Tolerated conc. (mg/L) | Source | Isolation context |
|---|---|---|---|---|
| ***P. simplicissimum*** | Pb | 11 000 | This study | Mine tailings (Zacatecas, Mexico) |
| ***P. lilacinus*** | Pb | 6 000 | This study | Mine tailings (Zacatecas, Mexico) |
| ***R. microsporus*** | Pb | 6 000 | This study | Mine tailings (Zacatecas, Mexico) |
| ***P. lilacinus*** | Cd | 950 | This study | Mine tailings (Zacatecas, Mexico) |
| ***R. microsporus*** | Cd | 550 | This study | Mine tailings (Zacatecas, Mexico) |
| ***F. oxysporum*** | Cd | 550 | This study | Mine tailings (Zacatecas, Mexico) |
| ***Cuninghamella sp.*** | Cd | 550 | This study | Mine tailings (Zacatecas, Mexico) |
| ***Penicillium sp.*** | Pb | 1 000 | Tian et al., 2019 [9] | Environmental isolate |
| ***P. lilacinus*** | Cd | 8 950 | Zeng et al., 2010 [42] | Cd smelting plant soil |
| ***Rhizopus microsporus*** | Pb | 250 | Oladipo et al., 2018 [50] | Mining site (Nigeria) |
| ***Mucor sp.*** | Cd | 20–30 | Deng et al., 2011 [35] | Metal-contaminated field |
| ***Aspergillus niger*** | Pb | ~500 | Tian et al., 2019 [9] | Environmental isolate |
| ***Trichoderma viride*** | Pb | ~750 | Luo et al., 2022 [31] | Adaptive exposure experiment |

Table S2. Comparison of $IC_{50}$ of heavy metals by filamentous fungi in this study and previous reports.

| **Fungal Species** | **Metal** | **$IC_{50}$ (mg/L)** | **Source** | **Isolation context** |
|---|---|---|---|---|
| ***P. simplicissimum*** | Pb | 3 874 | This study | Mine tailings (Zacatecas, Mexico) |
| ***P. lilacinus*** | Cd | 311 | This study | Mine tailings (Zacatecas, Mexico) |
| ***R. microsporus*** | Cd | 223 | This study | Mine tailings (Zacatecas, Mexico) |
| ***P. lilacinus*** | Cd | 29.25 | This study | Mine tailings (Zacatecas, Mexico) |
| ***R. microsporus*** | Cd | 25.18 | This study | Mine tailings (Zacatecas, Mexico) |
| ***F. oxysporum*** | Pb | 1 176 | This study | Mine tailings (Zacatecas, Mexico) |
| ***Cuninghamella sp.*** | Pb | 211.8 | This study | Mine tailings (Zacatecas, Mexico) |
| ***Paecilomyces variotii*** | Pb | 243 | Urquhart et al., 2022 [40] | Natural environments, controlled studies |
| ***Paecilomyces sp.*** | Cd | 10 | Urquhart et al., 2022 [40] | Natural environments, controlled studies |
| ***P. oxalicum*** | Pb | 1 243 | Văcar et al., 2021 [24] | Metal-contaminated soils from mining |
| ***Fusarium oxysporum*** | Pb | 1 568 | Văcar et al., 2021 [24] | Hg/Pb-contaminated soil |
